# A comprehensive review of the biodiversity of freshwater fish species in Valleys worldwide and in the Kingdom of Saudi Arabia

**DOI:** 10.5455/javar.2024.k784

**Published:** 2024-06-08

**Authors:** Tahani K. Altowairqi, Manal E. Shafi

**Affiliations:** 1 Department of Biological Sciences, Faculty of Science, King Abdulaziz University, Jeddah, Saudi Arabia; 2 Sustainable Agriculture Production Research Group, Department of Biological Sciences, Zoology, Faculty of Science, King Abdulaziz University, Jeddah, Saudi Arabia

**Keywords:** Biodiversity, freshwater, fish species, Valleys, KSA

## Abstract

An overview of freshwater fish variety worldwide and the variables influencing trends in variation between and within river basins are given in this review. Continental freshwater ecosystems are highly diverse and species-rich, housing nearly 18,000 species of fish (=50% of all fish species) in <0.5% of the total land area and providing a negligible (<0.01%) share of the planet’s water supply. Large lowland tropical river basins such as the Amazon, Congo, and Mekong basins are home to the greatest freshwater fish diversity. Freshwater species of fish depth variation at the global magnitude is correlated with the total amount and variation of aquatic habitats and the environment’s equilibrium overtime during the evolution of scales. The river continuum concept states that there is a predictable shift in fish species depth, diversity of species, and functional characteristics along gradients of environment from headwater to estuary. The ongoing trade of minerals and organic matter related to nearby floodplains is a strong factor in the number and variety of riverine fishes in most parts of the world (the flood pulse concept). Without coordinated conservation efforts, freshwater fishes will suffer significant losses in abundance and diversity due to the numerous threats they currently face worldwide. However, further development, adaptation, training, and guidance are needed. New technologies based on water conservation, suitable species, and local traditions are needed. Waste materials and local feed additives can also be used. Farmers should be provided with the necessary training and information.

## Introduction

The availability of water for consumption, hygiene, and watering are just a few of the many goods and services that freshwater ecosystems provide for human health [1]. Furthermore, although comprising less than 1% of the planet’s surface, freshwater ecosystems are exceptionally diverse, serving as home to roughly 30% of mammals and 10% of all referred-to living species [2]. The taxonomic environmental sustainability and functional variety of the social groups within a freshwater ecosystem are critical to the ecosystem’s functions and benefits to humans.

According to Reid et al. [3], freshwater habitats are highly diversified in biology, with over 140,000 species of microscopic fungi, plants, and animals found there, accounting for around 12% of all known species. There are over 18,000 different species of fish found in freshwater rivers, lakes, and wetlands, and hundreds more are discovered every year [4]. However, an environment that is under 0.5% of the entire territory of the earth and just roughly 0.3% of its hydrosphere’s capacity contains all of these unique evolutionary lineages [5]. Numerous species have an astounding array of biological, structural, and behavioral traits that enable them to live in nearly every aquatic setting on this continent.

Human impacts such as habitat change, water contamination, excessive fishing, and introduction of exotic species, river distractions, division and flow regulation, expansion of agricultural and urban landscapes, higher sea levels, and changed rainfall systems are posing a growing threat to the ecosystems of freshwater and their rich biodiversity [6,7]. The degree of riverine diversions and wetland degradation directly correlates with the decrease in freshwater fish [8]. Right now, the most endangered species groups on Earth are freshwater species, particularly amphibians and fishes [3].

The Arabian Peninsula has a low diversity of freshwater species due to the small volume of permanent water bodies, but this may be partly due to incomplete surveying. The region has a high diversity of aquatic plants, with 182 species represented, mostly in southern Arabia. Freshwater crabs and aquatic invertebrates are also species-poor, with only a few endemic species. Further studies are needed to establish a complete record of the region’s freshwater fauna. According to Darwall et al. [9], conserving every species in wetland ecosystems can be difficult, even for those with significant economic value. They proposed that it might be misguided to concentrate on commercially valuable species, particularly in the wadi systems of the Arabian Peninsula. Rather, they stressed the need for a cautious approach, considering the vital roles that all species play in the food webs and overall functioning of ecosystems.

This review provides a brief overview of freshwater fish diversity in Valleys globally, and the Kingdom of Saudi Arabia (KSA) highlights the importance of aquatic-terrestrial connections in preserving diverse and healthy fish groups, as well as the difficulties facing freshwater fish species. It also summarizes the key patterns and procedures involved in the biology and development of freshwater fish.

### Freshwater habitats (Valleys)

A well-known and highly valued component of fisheries’ biological processes, administration, and preservation is habitat [10]. Fisheries administrations across North America fund programs to inventory, monitor, and improve aquatic habitats to assess, preserve, and enhance fish populations [11]. In fact, under the direction of qualified experts, numerous citizen groups are involved in restoration and improvement initiatives because the importance of habitat in fisheries is so widely recognized. Several prominent habitat improvement initiatives that are elements of fisheries management include riparian management [12], manufactured reef-building, and stream rehabilitation [13]. Nevertheless, this kind of administration can be challenging and necessitates a thorough comprehension of how habitat affects aquatic ecosystem function.

Although managing fish habitat is widely acknowledged in fisheries biology, there is a great deal of variation in how it is incorporated into management. Though it has proven more challenging to identify the precise causes of degraded conditions or create predictive models helpful for habitat rehabilitation, broad principles of habitat quality have created beneficial biotic indicators that measure habitat quality [14]. We remain in the early stages of quantifying fish population reactions to changes in habitat that could be important for repair and improvement initiatives and helpful in determining limitations. It can be challenging to pick out, out of the many important habitat elements in aquatic systems, just one that might restrict a fish population. Therefore, it is necessary to observe population-level reactions to administration.

**Figure 1.**
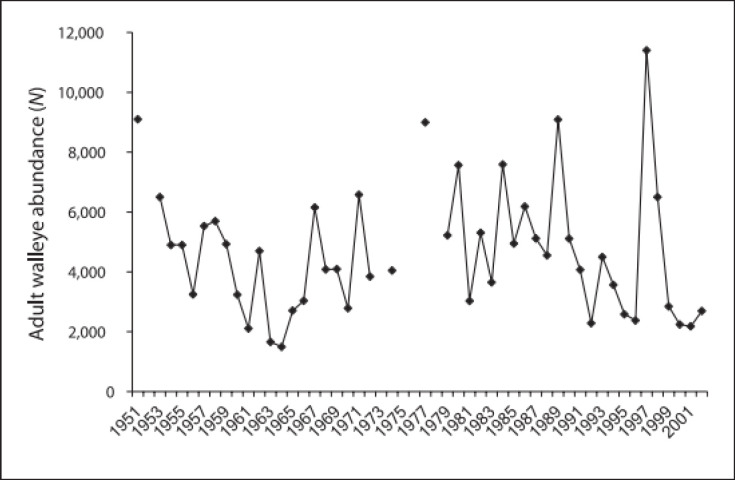
The Walleye population levels in Wisconsin’s Escanaba Lake from 1951 to 2002 based on creel surveys. The lake is situated in the Northern Highland State Forest, which is under protection. It is thought that the natural environment has not changed significantly throughout this time.

Furthermore, habitat is only one aspect of ecosystems; environmentally friendly, weather-related, and social interactions can frequently conceal or overrule the population’s reactions to habitat, which, in many instances, can appear to be restricting. Examples of these interactions include efficiency and predator-prey behavior. For instance, dependent on density year-class thicknesses (Fig. 1) caused significant annual variations in adult walleye wealth in Escanaba Lake, Wisconsin (1951–2002), but the physical habitat—which includes the amount of water, materials in the littoral zone, water quality, and the presence and abundance of macrophytes—remained mostly consistent. Therefore, lake fish populations could not shift noticeably beyond the amount reflecting organic variation if the habitat is directly altered [15].

**Figure 2.**
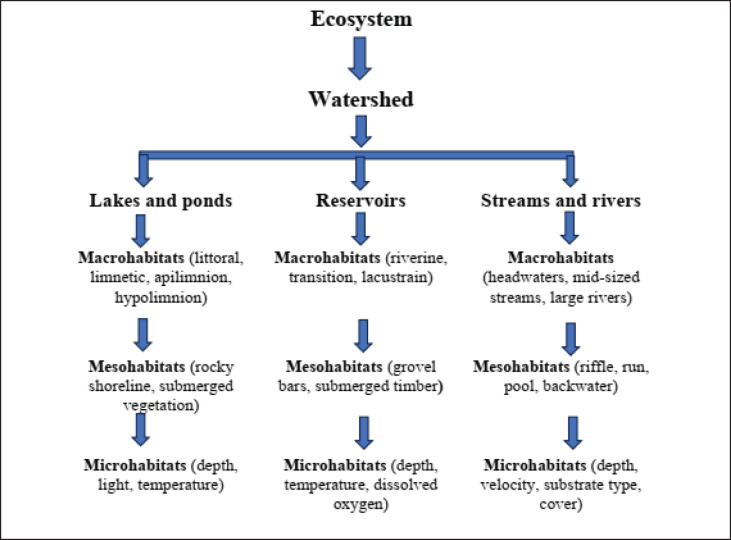
Spatial hierarchy of terms related to aquatic habitats frequently employed in fisheries projects.

Fish adjust to their fundamental dynamics, resulting in diverse sizes, shapes, chemistry, and positions in aquatic environments. The habitats in these systems vary and range in size from tiny springs to huge lakes with different nutrient levels. As seen in Figure 2, these habitats are arranged in a hierarchy of spatial scales, with ecosystems enclosed by watersheds.

Faghihinia et al. [16] reported using the SCI-E and SSCI databases of the Web of Science to investigate global freshwater ecological diversity patterns over the previous 20 years. It was discovered that studies on river and lake ecosystems were overrepresented in developed areas. The investigation additionally identified which environmental driving factors—such as invasive species, eutrophication, land use change, climate change, and habitat heterogeneity and degradation—have received the least attention. The study also discovered that taxonomic groups were being studied in excess, which implies that more advancement in freshwater biodiversity can be made by refocusing research attention on less established but important emerging themes.

### Biodiversity of freshwater fishes

Like a family or species of fish, the variety of a clade is a macro-evolutionary equilibrium among variation rates, which boosts variety and extinction, which lowers diversity. It has been proposed that fish speciation rates are greater in lacustrine habitats than in river habitats [17]. However, speciation at the allopatric level inside the dendritic spatial arrangement of continental rivers is believed to be responsible for most freshwater fish diversity [17,18]. As a result of river capture and sea level fluctuations, river basins have experienced more complex historical habitat rearrangements than lakes. They also have a far larger total habitat footprint [19,20].

As evidenced by global weather cycles throughout the Pliocene and Pleistocene, saltwater tolerances have an important effect on fish biogeographic variations and natural histories, leading to marine violations and regression models. Due to rising sea levels, river mouths have become geographically isolated from other coastal drainages. This causes vicarious incidents for elementary freshwater fish species and semipermeable filter obstacles for additional and outside freshwater fish species. According to Leprieur et al. [21] and Abreu et al. [22], decreasing sea levels and retreating shorelines also combine the mouths of coastal rivers, permitting species to disperse (extend their geographic range) and reducing the likelihood of extinction.

Variations in salt tolerance also shed light on the history of freshwater creatures found in isolated areas. Primary freshwater fish are typically shared by the mainland and continental islands, such as Trinidad or Britain, with a geological history of being connected to an adjacent landmass. Though they have never been joined to a continent, oceanic islands such as Iceland or Hawaii lack native primary freshwater fish. Oceanic islands typically have secondary or auxiliary fish species.

**Figure 3.**
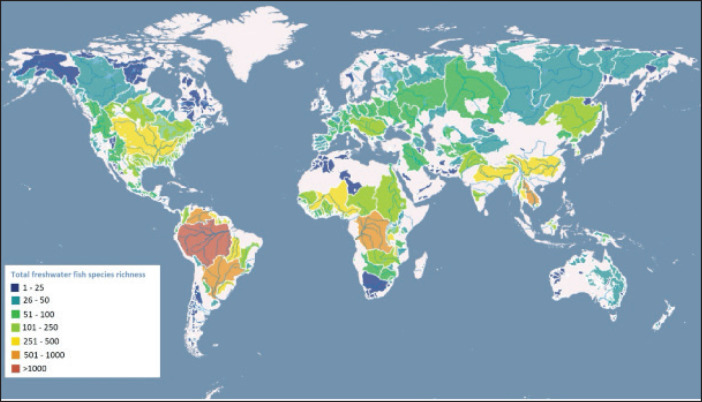
Global freshwater fish richness patterns (rivers basins as spatial units). Map from Tedesco, Jézéquel, and Oberdorff [26], global freshwater fish species richness; accessed through the global freshwater biodiversity atlas.

Large tropical river and lake basins such as the Amazon, Congo, and Mekong, in addition to large ancient lakes like those in the East African Rift Valley, exhibit the highest levels of freshwater fish diversity (Fig. 3). Compared to continental regions of equivalent size, Islands typically have a lower proportion of elementary freshwater fish and less variety of freshwater fish. The three main factors influencing the variation of riverine fish species depth worldwide are the overall quantity of aquatic habitat, the variation in the spatial distribution of aquatic habitats, and the constancy of aquatic settings over natural time scales. Since these elements are interconnected, it is challenging to determine how much each contributes within a given area.

Habitat area, where the species-discharge relationship results from the interaction between the overall quantity of aquatic habitat and the size of river basins [23]. More species are generally found in greater river basins than in lesser-sized ones. When all else is equal, fish populations in larger drainage basins are greater than those in smaller ones, lowering the likelihood of extinction following severe disturbance incidents. Additionally, compared to smaller rivers, larger river basins typically have more interest niches that can be utilized due to their greater habitat variety.

Strategies of habitat variation and geographic revenue have an upward trend with the number of fish species [24]. The diverse distribution of freshwater habitats throughout biogeographic space and evolutionary time is a primary factor contributing to the exceptional diversity of species found in freshwater [20]. Fisheries populations have numerous opportunities to disperse, which lowers their risk of extinction [25], and to grow geographically separated, which increases the likelihood of speciation. These chances are made possible by the distinct geographic conditions found in riverine and lacustrine environments. In contrast, most marine environments exhibit high levels of connectivity, with oceanic groups of organisms generally exhibiting lower rates of descent buildup [17].

For the fish of the Arabian Peninsula, a fair amount of taxonomic study has been conducted, but the taxonomy of certain species remains unclear. Three Arabian cyprinids, for instance, are frequently assigned to the genus Barbus. It has been known for many years that *B.* *arabicus*, the third species, is not a member of the *Barbus* genus. Borkenhagen [27] has placed this species in the new genus *Arabibarbus.*

According to Alharthi [28], freshwater fish are primarily found in sewers in the southwest of the Kingdom, in both the highlands and lowlands of the Sarawat Mountain Range (eastern and western drainage systems). This study found that non-native fish species, including *Oreochromis spp*. and *Carassius spp*., are present in the Al Baha Region, Abha, and Rabigh dams. This indicates that non-native fish species invade western and eastern drainage systems. In eight water bodies in Saudi Arabia, the study looked at the ecology of three native and four non-native species. Three fish seasons were harvested, and their age, growth, feeding ecology, and reproductive traits were assessed. The results indicated that habitat type and environmental quality were the main factors influencing growth rates.

Freshwater fish are vital to Saudi Arabia’s wetland biodiversity [29]. Data accessibility regarding these kinds of organisms is a major challenge for ecological management professionals. It is frustrating to lack knowledge about threats and their biological and ecological characteristics. Based on previously released payments, Hamidan and Shobrak [30] surveyed Saudi Arabia’s freshwater fish in 22 distinct locations between April and May 2013 and visited new sites. All previously known species were confirmed to be present, except for *Carasobarbus apoensis*. About thirty years ago, *Acanthobrama hadiyahensis* was described, and then it was first documented. They concluded that *Garra buettikeri* is classified as vulnerable and *Acanthobrama hadiyahensis* as a critically endangered species, with the remaining species being of the least concern. The distribution of certain freshwater fish species in the KSA is displayed in Table 1, focusing on species brought in for biological management and seen in man-made lakes.

**Figure 4.**
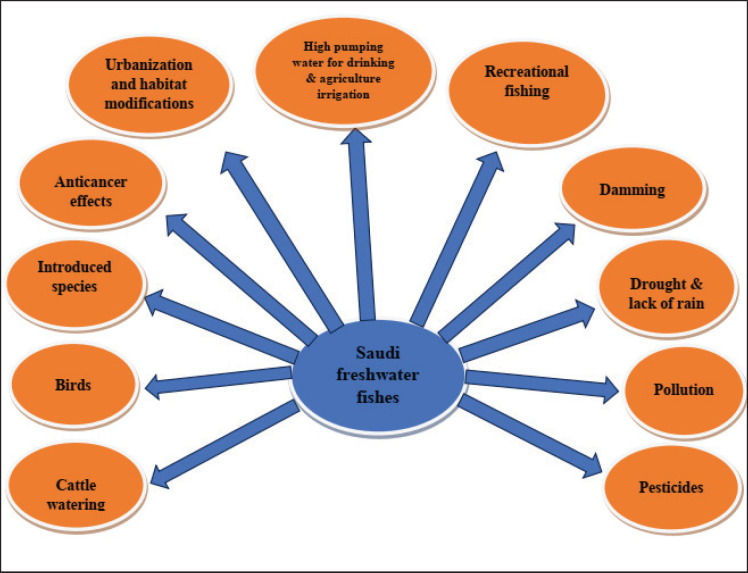
Surveys conducted in Saudi wetlands revealed possible threats to freshwater fish populations (pers. obs.).

Freshwater fish in Saudi Arabia face multiple threats from operations with both high and low effects. To address or lessen their potential or actual impacts, these threats should be assessed using particular scientific frameworks to ascertain their mechanisms and effects. This will aid in developing an efficient management strategy for Saudi Arabia’s freshwater fishery. The possible hazards and pressures noted during data collection visits to Saudi Arabian dams and wadis are depicted in Figure 4. More information about a few of the major pressures and their effects is given in the remaining portion of this subsection. Other sources, such as EPAA [31], Al-Kahem [32], Hamidan and Aloufi [33], and Freyhof et al. [34], also address threats to Saudi freshwater fish.

### The commercial importance of some species

For many global populations, especially in developing nations, fish is healthier than meat, poultry, and eggs. Fish offers relatively inexpensive and easily accessible protein sources and long chains of n-3 fatty acids, amino acids, vitamins, and minerals, which help provide healthier choices for a well-rounded dietary intake. Fish’s protein content typically ranges from 15% to 20% on average [37].

**Table 1.** The freshwater fish species that were brought to Saudi Arabia [32–35].

Family	Species/group	Purpose	References
Poeciliidae	*Gambusia affinis* (Baird & Girard, 1853)	Biological control	[32]
	*Poecilia latipinna* (Lesueur, 1821)		[36]
	*Poecilia reticulata Peters*, 1859		[32]
	*Xiphophorus maculatus* (Günther, 1866)		[32]
Cichlidae	*Oreochromis aureus* (Steindachner, 1864)	Aquaculture	[32]
	*Oreochromis mossambicus* (Peters, 1852)	Aquaculture	
	*Oreochromis niloticus* (Linnaeus, 1758)	Aquaculture Biological control	
	*Oreochromis spilurus* (Günther, 1894)	Aquaculture & research	
Clariidae	*Clarias gariepinus* (Burchell, 1822)	Aquaculture Biological control	[32]
Cyprinidae	*Carassius auratus* (Linnaeus, 1758)		
	Ornamental fish	Pet purposes	
	*Ctenopharyngodon idella* (Valenciennes, 1844)	Biological control (weed control)	[35]
	*Cyprinus carpio Linnaeus*, 1758	Biological Control & research & aquaculture	
Acipenseridae	*Acipenser gueldenstaedtii* Brandt & Ratzeburg, 1833	Aquaculture (Caviar)	
	*Acipenser baerii* Brandt, 1869		

Many people, particularly those who live near rivers, rely on freshwater fish species for food, survival, and additional revenue [38]. According to Fawole et al. [39], fish and fishery products are an important source of essential nutrients for varied and healthful diets. Fish, with some exceptions for particular species, is typically low in cholesterol, carbohydrates, and saturated fats. Along with high-quality protein, fish offers a variety of important micronutrients, such as omega-3 fatty acids (Eicosapentaenoic acid and Docosahexaenoic acid) and multiple vitamins (A, B, and D), as well as minerals (calcium, iodine, zinc, iron, and selenium). Tryptophan, cysteine, methionine, lysine, and threonine are essential amino acids abundant in fish protein [40]. Essential amino acids are considered crucial to human health and nutritional advancement [41]. Research has shown that eating fish positively affects cardiovascular disease, stroke, age, muscular deterioration, and psychological well-being [42]. Additionally, there is strong proof of the advantages of advancement and growth, especially for women during pregnancy and young children during infancy, for the best possible brain growth [43].

Globally, greetings of invasive alien species have increased due to the expansion of international trade [44]. Since they are the second leading cause of global species extinction, biological incursions are now acknowledged as posing significant risks to ecological diversity [45]. According to Gozlan [46], non-native freshwater fish have become established in every biogeographical world, making them one of the most established species. The effects of creating non-native aquatic fish species are varied. They can include local hybridization with native fauna, as in the case of native west slope competitive fish (*Oncorhynchus clarkii lewisi*) and invasive brook trout (*Oncorhynchus mykiss*) in Canadian rivers, or major shifts in the arrangement of species that result in taxonomic mixing over large regions, like the Laurentian Great Lakes [47].

However, not every non-native fish species that arrives establishes itself or has an effect. Prior research has demonstrated that three types of factors affect each stage of the assault process: traits specific to the organisms, ecological traits of the host or host ecosystem, and socioeconomic traits. Non-native fish species with a wide range of food sources are easier to establish regarding species traits than are more specific species. Like ecological traits, the development and spread of non-native species are influenced by the extent of their native range [48]. In terms of socioeconomic traits, it is well known that certain introduction routes are a significant source of new freshwater fish introductions and businesses like fish farming and the ornamental trade [49].

### Aquaculture and its importance in preserving the species

Worldwide population explosion and increasing appetites for food are driving the swift growth and amplification of agricultural land [50,51]. These elements, along with rising transpiration and falling precipitation, could also be causing deforestation, as reported by the United Nations Convention to Combat Desertification [52] and the United Nations University, Institute for Water, Environment, and Health. Water is necessary for humans to survive in semi-arid and arid desert areas. Farmers who engage in dry-land agriculture are particularly impacted. Farmers typically plant barley, wheat, or other staple grains throughout the period when it rains. Grain harvesting and storage occur in the months following the rainy season, provided there is enough rainfall (more than 200–400 mm). The farmer and his loved ones receive meat and milk, which are significant forms of animal protein, from the cows and sheep they feed on crop residue and weeds [53]. Yields from crops and grazing land may not be enough to meet a family’s survival needs in years with less precipitation, particularly if a series of droughts occur.

Aquaculture has grown in importance to provide fish protein to feed the expanding human population in areas where other demanding farming methods are prohibitively expensive or unfeasible [40]. The environment and climate, especially the lower annual precipitation, significantly hinder traditional farming and demanding livestock methods in semi-arid and arid areas [54]. Fish might be effectively raised in marine or brackish waters, according to experiments conducted on desert fish farming between 1963 and 1965 [55]. Fish can find appropriate and beneficial food in these waters due to their high minerals, elevated temperatures, and high sunlight [56]. Aquaculture activities have widened to new borders, such as subjected and faraway seas, or hostile areas, such as arid regions and deserts, where contemporary sustainable aquaculture methods may be utilized more effectively. This is due to the rising rivalry for water and land in many different economic sectors [57].

Commerce, job opportunities, and fish farming are all correlated. The growing need for fish and other seafood is causing a rapid transformation in the aquaculture and fishing sectors, which employ roughly 600 million people. Increased living standards, development, postharvest procedures, and shipping modifications, and dietary trends emphasizing improved nutrition and health are all predicted to contribute to a rise in use. The most recent data showed that women made up about 21% of the workforce in this industry. Building resilience is crucial for fair and equitable growth [58].

Regardless of the COVID-19 pandemic, the generation of aquatic animals for human consumption directly exceeded 157 million metric tons in 2020. In nations with lower incomes, the percentage of animal proteins obtained from aquatic foods was 23% in 2019, while in some regions of Asia and Africa, it was over 50%. Asia accounted for 70% of global aquatic animal consumption from fisheries and aquaculture in 2020, with ‘’the Americas, Europe, Africa, and Oceania closely behind. China, Indonesia, Peru, the Russian Federation, the United States, India, and Vietnam’’ were among the top fisheries manufacturers in that order [58].

One-fifth of the earth’s surface comprises arid regions [59], and deforestation and global warming are anticipated to accelerate salinity. Despite their difficulties, arid regions have an opportunity to grow thanks to technological advances in arid aquaculture. Ocean water can be utilized in fish cultivation and establish farming by growing organisms that can withstand salt content, and fish farming can be done in desert saline or brackish waters [60]. Fish are given an appropriate and beneficial food base because of the quantity of minerals in this water and the high outside temperatures and solar radiation. Because then, researchers have worked on developing methods that would be appropriate for fish farming. Numerous nations have created effective models for raising fish in salty or brackish water below deserts [61]. Proper water management, such as reusing and conservation, is crucial in such systems. Additionally, contemporary aquaculture technologies such as aquaponics [62], biofloc [63], incorporated multitrophic fish farming [64], circulatory systems for aquaculture, and aqua mimicry [65] should be implemented.

Aquaculture’s cooperation with cultivation is making it more and more appealing in regions with scarce water resources. These systems enable fresh vegetable products and high-quality fish protein production using less water than they would if run separately [55].

### Efforts in KSA to raise awareness about important fish consumption, especially in mountain and desert regions

Globally, wetland areas and aquatic ecosystems benefit the natural world and society, but they are threatened by overexploitation, particularly in Saudi Arabia. These organizations offer hunting, fishing, tourism, agriculture, and raising animals. Alharthi [28] notes that certain activities, such as water abstraction, agriculture, and effluent discharges, are degrading them.

According to Khan et al. [66], to increase the public’s curiosity about fish farming, appropriate policies should be developed to inform people about hydroponics, or fish farming, and its significance via fisheries outreach programs. As per the Food and Agriculture Organization, an average yearly increase of 1.62% from 2001 to 2011 was observed in the worldwide per-person seafood intake, which amounted to roughly 18.9 kg in 2011. Many different societies, encompassing urban and rural regions, adhere to a rise in the rate of fishing [67]. Fish is an important source of protein, vitamins, and other nutrients essential to human well-being [68]. In the world, fish account for 6.5% of all protein consumed for human consumption and 16.6% of the supply of animal protein [69]. Aquaculture and fish farming are major contributors to improving the economic situation worldwide because they create jobs. Between 660 and 880 million people rely entirely or partially on aquaculture for their income [70].

The growth of fish farming in arid environments, like desert regions, requires implementing contemporary aquaculture methods, like recirculation systems and manufacturing technologies that emphasize water management techniques, especially when dense aquaculture is practically and financially feasible. Cultivating fish in up to 50 kg/m^3^ of water usually requires a small area and is incredibly efficient regarding water usage [71]. The aquaculture industry can help combat the decline in food security. Surpassing earthly meat manufacturing and capture fisheries, this sector is the most rapidly growing source of animal food worldwide [72].

Saudi Arabia covers 2.15 million km^2^ and is a dry country with few freshwater resources. Situated in a continent-wide zone with scorching summers and low winter conditions, the Kingdom resembles a desert. It is also distinguished by little annual precipitation and the absence of permanent ponds or rivers. Both the climate and the depletion of subsurface water resources present ongoing challenges. Due to a severe shortage, water remains a precious asset and holds the top spot among the Kingdom’s assets. Despite being an endless resource, society has very little access to water. The current scenario puts a great deal of strain on the water resources that are currently available because of the increasing number of people and increasing standards of living in civil society. The lack of freshwater indicates that fish farming, particularly those conducted along the Red Sea coast, is the way to meet Saudi Arabia’s future needs for fish food.

With a population of about 30 million, Saudi Arabia is the most populous nation in the Gulf Cooperation Council. Its long coast and pleasant water inlets make it an ideal location for aquaculture. Due to the perfect environment for fish farming, aquaculture was first established in the Kingdom in the 1980s, after the Ministry of Agriculture realized its significance [73]. Although fresh fish is not a staple food in Saudi Arabia, its demand is growing. The amount of fish consumed annually per person increased from 3 kg in 1977 to 6.5 kg in 1998 and almost 8 kg in 2007. Just 11.5 kg were supplied per person in 2010 [69].

## Conclusion

Focusing on global freshwater ecosystems that are incredibly varied and species-rich, this assessment summarizes the variety of freshwater fish globally. The most diverse basins of rivers are large lowland regions, such as the Amazon, Congo, and Mekong, which are tropical. According to the river continuum theory, fish species diversity and depth will inevitably change as environmental variations change. The flood pulse idea shows how continuing trade affects fish living in rivers. Conservation measures are required to avoid a major decline in the diversity and abundance of freshwater fish.

## List of abbreviations

KSA, Kingdom of Saudi Arabia.

## References

[1] Green PA, Vörösmarty CJ, Harrison I, Farrell T, Sáenz L, Fekete BM (2015). Freshwater ecosystem services supporting humans: pivoting from water crisis to water solutions. Glob Environ Change.

[2] Winemiller KO (2018). Trends in biodiversity: freshwater. Encycloped anthropocene..

[3] Reid AJ, Carlson AK, Creed IF, Eliason EJ, Gell PA, Johnson PT (2019). Emerging threats and persistent conservation challenges for freshwater biodiversity. Biol Rev Camb Philos Soc.

[4] Fricke R, Eschmeyer WN, Van der Laan R (2020). Eschmeyer’s catalog of fishes: genera, species, references. http://researcharchive.calacademy.org/research/ichthyology/catalog/fishcatmain.asp.

[5] Lundberg JG, Kottelat M, Smith GR, Stiassny ML, Gill AC (2000). So many fishes, so little time: an overview of recent ichthyological discovery in continental waters. Ann Missouri Bot Gard.

[6] Dudgeon D (2019). Multiple threats imperil freshwater biodiversity in the anthropocene. Curr Biol.

[7] Díaz S, Settele J, Brondízio ES, Ngo HT, Guèze M, Agard J, IPBES (2019). Summary for policymakers of the global assessment report on biodiversity and ecosystem services of the Intergovernmental Science-Policy Platform on Biodiversity and Ecosystem Services.

[8] Albert JS, Destouni G, Duke-Sylvester SM, Magurran AE, Oberdorff T, Albert JS (2021). Scientists’ warning to humanity on the freshwater biodiversity crisis. Ambio.

[9] Darwall W, Tweddle D, Smith K, Skelton P (2009). The status and distribution of freshwater biodiversity in southern Africa.

[10] Naiman RJ, Latterell JJ (2005). Principles for linking fish habitat to fisheries management and conservation. J Fish Biol.

[11] Fisher WL, Burroughs JP (2003). Stream fisheries management in the United States: a survey of state agency programs. Fisheries.

[12] Naiman RJ, Bilby RE, Bisson PA (2000). Riparian ecology and management in the Pacific coastal rain forest. BioScience.

[13] Alexander GG, Allan JD (2006). Stream restoration in the Upper Midwest, USA. Restor Ecol.

[14] Stoneman CL, Jones ML (2000). The influence of habitat features on the biomass and distribution of three species of southern *Ontario stream salmonines*. Trans Am Fish Soc.

[15] Neuswanger D, Bozek MA (2004). Preliminary assessment of effects of rock habitat projects on walleye reproduction in 20 northern Wisconsin lakes: a summary of case histories as of March 2004.

[16] Faghihinia M, Xu Y, Liu D, Wu N (2021). Freshwater biodiversity at different habitats: research hotspots with persistent and emerging themes. Ecol Indic.

[17] Miller EC (2020). Comparing speciation rates in lakes, rivers, and the sea. BioRxiv.

[18] Albert JS, Petry P, Reis RE, Albert JS, Reis RE (2011). Major biogeographic and phylogenetic patterns. Historical biogeography of neotropical freshwater fishes.

[19] Albert JS, Crampton WGR (2010). The geography and ecology of diversification in neotropical freshwaters. Nat Educ Knowl.

[20] Albert JS, Craig JM, Tagliacollo VA, Petry P (2018). Upland and lowland fishes: a test of the river capture hypothesis. Mountains, Climate Biodivers.

[21] Leprieur F, Tedesco PA, Hugueny B, Beauchard O, Dürr HH, Brosse S (2011). Partitioning global patterns of freshwater fish beta diversity reveals contrasting signatures of past climate changes. Ecol Lett.

[22] Abreu JMS, Saraiva ACS, Albert JS, Piorski NM (2020). Paleogeographic influences on freshwater fish distributions in northeastern Brazil. J S Am Earth Sci.

[23] Abd El-Hack ME, El-Saadony MT, Ellakany HF, Elbestawy AR, Abaza SS, Geneedy AM (2022). Inhibition of microbial pathogens in farmed fish. Mar Pollut Bull.

[24] Alagawany M, Taha AE, Noreldin A, El-Tarabily KA, Abd El-Hack ME (2021). Nutritional applications of species of *Spirulina* and *Chlorella* in farmed fish: a review. Aquaculture.

[25] Miller E, Román-Palacios C (2019). Evolutionary time explains the global distribution of freshwater fish diversity. BioRxiv.

[26] Tedesco PA, Oberdorff T, Cornu JF, Beauchard O, Brosse S, Dürr HH (2013). A scenario for impacts of wateravailability loss due to climate change on riverine fish extinctionrates. J Appl Ecol.

[27] Borkenhagen K (2014). A new genus and species of cyprinid fish (Actinopterygii, Cyprinidae) from the Arabian Peninsula, and its phylogenetic and zoogeographic affinities. Environ Biol Fishes.

[28] Alharthi IGZ (2019). Conservation of freshwater fishes in Saudi Arabia. Doctoral Dissertation, University of Hull, Hull, UK.

[29] SWA (2008). Saudi Arabian National Biodiversity Assessment (SAMBA), status report and assessment plan. https://www.cbd.int/doc/world/sa/sa-nbsap-01-en.pdf.

[30] Hamidan NAF, Shobrak M (2019). An update on freshwater fishes of Saudi Arabia. Jordan J Biol Sci.

[31] EPAA (2002). Conservation Assessment and Management Plan (CAMP) for the Threatened Fauna of Arabia’s Mountain Habitat.

[32] Al-Kahem H (2004). Fish diversity in Saudi Arabia (in Arabic). Ichthyology.

[33] Hamidan N, Aloufi AA (2014). Rediscovery of *Acanthobrama hadiyahensis* (Cyprinidae) in Saudi Arabia. J Fish Biol.

[34] Freyhof J, Hamidan NA, Feulner GR, Harrison I, García N, Harrison I, Cox N, Tognelli MF (2015). The status and distribution of freshwater fishes of the Arabian Peninsula. The status and distribution of freshwater biodiversity in the Arabian peninsula.

[35] Abd El-Hack ME, Alagawany M, Farag M, Tiwari R, Karthik K, Dhama K (2016). Beneficial impacts of thymol essential oil on health and production of animals, fish and poultry: a review. J Essent Oil Res.

[36] Abd El-Hack ME, Abdelnour SA, Khafaga AF, Taha AE, Abdel-Latif HM, Hassanien MFR (2021). *Nigella sativa* seeds and its derivatives in fish feed. Black cumin (*Nigella sativa*) seeds: chemistry, technology, functionality, and applications.

[37] Food and Agriculture Organization (2010). Nutritional elements of fish. http://http://www.fao.org/fishery/topic/12319/en.

[38] Alagawany M, Abd El-Hack ME, Farag MR, Shaheen HM, Abdel-Latif MA, Noreldin AE (2020). The applications of and its derivatives in human, ruminant and fish nutrition–a review. Ann Anim Sci.

[39] Fawole OO, Ogundiran MA, Ayandiran TA, Olagunju OF (2007). Proximate and mineral composition in some selected fresh water fishes in Nigeria. Internet J Food Saf.

[40] FAO (2011). FAO fisheries and aquaculture report No 978.

[41] Limin L, Feng X, Jing H (2006). Amino acids composition difference and nutritive evaluation of the muscle of five species of marine fish, *Pseudosciaena crocea* (large yellow croaker), *Lateolabrax japonicus* (common sea perch), *Pagrosomus major* (red seabream), *Seriola dumerili* (*Dumeril’s amberjack*) and *Hapalogenys nitens* (black grunt) from Xiamen Bay of China. Aquac Nutr.

[42] Peet M, Stokes C (2005). Omega-3 fatty acids in the treatment of psychiatric disorders. Drugs.

[43] Young G, Conquer J (2005). Omega-3 fatty acids and neuropsychiatric disorders. Reprod Nutr Dev.

[44] Seebens H, Blackburn TM, Dyer EE, Genovesi P, Hulme PE, Jeschke JM (2017). No saturation in the accumulation of alien species worldwide. Nat Commun.

[45] Naiel MA, Shehata AM, Negm SS, Abd El‐Hack ME, Amer MS, Khafaga AF (2020). The new aspects of using some safe feed additives on alleviated imidacloprid toxicity in farmed fish: a review. Rev Aquac.

[46] Gozlan RE (2008). Introduction of non‐native freshwater fish: is it all bad?. Fish Fish.

[47] Naiel MA, Alagawany M, Patra AK, El-Kholy AI, Amer MS, Abd El-Hack ME (2021). Beneficial impacts and health benefits of macroalgae phenolic molecules on fish production. Aquaculture.

[48] Marchetti MP, Moyle PB, Levine R (2004). Alien fishes in California watersheds: characteristics of successful and failed invaders. Ecol Appl.

[49] Gozlan RE, Britton JR, Cowx I, Copp GH (2010). Current knowledge on non‐native freshwater fish introductions. J Fish Biol.

[50] Golden CD, Koehn JZ, Shepon A, Passarelli S, Free CM, Viana DF (2021). Aquatic foods to nourish nations. Nature.

[51] Mehana ESE, Khafaga AF, Elblehi SS, Abd El-Hack ME, Naiel MA, Bin-Jumah M (2020). Biomonitoring of heavy metal pollution using acanthocephalans parasite in ecosystem: an updated overview. Animals.

[52] United Nations Convention to Combat Desertification (UNCCD) (2007). Desertification, exacerbated by climate change, represents one of the greatest environmental challenges of our times. UNCCD thematic fact sheet series No. 1. Climate Change and Desertification.

[53] Abd El-Hack ME, El-Saadony MT, Nader MM, Salem HM, El-Tahan AM, Soliman SM (2022). Effect of environmental factors on growth performance of Nile tilapia (*Oreochromis niloticus*). Int J Biometeorol.

[54] Cowan N, Ferrier L, Spears B, Drewer J, Reay D, Skiba U (2022). CEA systems: the means to achieve future food security and environmental sustainability?. Front Sustain Food Syst.

[55] Crespi V, Lovatelli A (2011b). Global desert aquaculture at a glance. Aquaculture in desert and arid lands. Fisheries and Aquaculture Circular No. 1195.

[56] Crespi V (2009). Support to the development of desert aquaculture and management of the brackish water lakes in Algeria. FAN-FAO Aquac Newslett.

[57] Pueppke SG, Nurtazin S, Ou W (2020). Water and land as shared resources for agriculture and aquaculture: insights from Asia. Water.

[58] FAO (2022). Record fisheries and aquaculture production makes critical contribution to global food security [EN/AR/IT/RU/ZH].

[59] Smith M, Veth P, Hiscock P, Wallis LA, Veth P, Smith S, Hiscock P (2005). Global deserts in perspective. Desert peoples: archaeological perspectives.

[60] Cabrera-González M, Ramonet F, Harasek M (2022). Development of a model for the implementation of the circular economy in desert coastal regions. Land.

[61] Crespi V, Lovatelli A, Appelbaum S, Hulata G, Karimov B, Kolkovski S Aquaculture in desert and arid lands: development constraints and opportunities: FAO Technical Workshop, Hermosillo, Mexico, 6-9 July 2010.

[62] Kotzen B, Emerenciano MGC, Moheimani N, Burnell GM, Goddek S, Joyce A, Kotzen B, Burnell GM (2019). Aquaponics: alternative types and approaches. Aquaponics food production systems: combined aquaculture and hydroponic production technologies for the future.

[63] Khanjani MH, Sharifinia M, Emerenciano MGC (2023). A detailed look at the impacts of biofloc on immunological and hematological parameters and improving resistance to diseases. Fish Shellfish Immunol.

[64] Khanjani MH, Zahedi S, Mohammadi A (2022b). Integrated multitrophic aquaculture (IMTA) as an environmentally friendly system for sustainable aquaculture: functionality, species, and application of biofloc technology (BFT). Environ Sci Pollut Res.

[65] Khanjani MH, Mozanzadeh MT, Fóes GK (2022a). Aquamimicry system: a sutiable strategy for shrimp aquaculture–a review. Ann Anim Sci.

[66] Khan AQ, Aldosari F, Hussain SM (2018). Fish consumption behavior and fish farming attitude in Kingdom of Saudi Arabia (KSA). J Saudi Soc Agric Sci.

[67] Bienenfeld LA, Golden AL, Garland EJ (2003). Consumption of fish from polluted waters by WIC participants in East Harlem. J Urban Health.

[68] Sun YHC (2008). Health concern, food choice motives, and attitudes toward healthy eating: the mediating role of food choice motives. Appetite.

[69] FAO (2013). Fishery and aquaculture statistics yearbook.

[70] Allison EH, Delaporte A, Hellebrandt de Silva D (2013). Integrating fisheries management and aquaculture development with food security and livelihoods for the poor. Report Submitted to the Rockefeller Foundation, School of International Development, University of East Anglia, Norwich, UK.

[71] Kolkovski I, Simon Y, Hulata G, Kolkovski C, Ayaril N, Lucas JS, Southgate PC (2013). Desert aquaculture. Aquaculture: farming aquatic animals and plants.

[72] FAO (2002). The state of world fisheries and agriculture.

[73] Essa Al-Sunaiher A, FAO (2010). National aquaculture sector overview. Kingdom of Saudi Arabia. National aquaculture sector overview fact sheets.

